# Massive Stokes shift in 12-coordinate Ce(NO2)_6_^3−^: crystal structure, vibrational and electronic spectra

**DOI:** 10.1038/s41598-018-34889-4

**Published:** 2018-11-08

**Authors:** Yuxia Luo, Chun-Kit Hau, Yau Yuen Yeung, Ka-Leung Wong, Kwok Keung Shiu, Peter A. Tanner

**Affiliations:** 1 Department of Chemistry, Hong Kong Baptist University, 224 Waterloo Road, Kowloon, Hong Kong, S.A.R. P. R. China; 2 Department of Science and Environmental Studies, The Education University of Hong Kong, 10 Lo Ping Road, Tai Po, New Territories, Hong Kong, S.A.R. P. R. China

## Abstract

The Ce^3+^ ion in Cs_2_NaCe(NO_2_)_6_ (**I**), which comprises the unusual *T*_*h*_ site symmetry of the Ce(NO_2_)_6_^3−^ ion, demonstrates the largest Ce-O Stokes shift of 8715 cm^−1^ and the low emission quenching temperature of 53 K. The activation energy for quenching changes with temperature, attributed to relative shifts of the two potential energy curves involved. The splitting of the Ce^3+^ 5d^1^ state into two levels separated by 4925 cm^−1^ is accounted for by a first principles calculation using the crystal structure data of **I**. The NO_2_^−^ energy levels and spectra were investigated also in Cs_2_NaLa(NO_2_)_6_ and modelled by hybrid DFT. The vibrational and electronic spectral properties have been thoroughly investigated and rationalized at temperatures down to 10 K. A comparison of Stokes shifts with other Ce-O systems emphasizes the dependence upon the coordination number of Ce^3+^.

## Introduction

Hexanitrito complexes of transition metals, such as [TM(NO_2_)_6_]^4−^ TM = Cu^2+^, Co^2+^, exhibit Jahn-Teller distortion of the TM-N_6_ octahedron^1^. It was unexpected^2^ that the analogous complexes of lanthanide ions (Ln^3+^) exhibit a different coordination geometry, with the ligand oxygen rather than nitrogen being coordinated to Ln^3+^. The [Ln(NO_2_)_6_]^3−^ moiety has the 12-coordinated Ln^3+^ situated at a site of the novel *T*_*h*_ point group symmetry^3–5^. The magnetic properties^6^ of this series showed some similarities, but also unique differences, from those of the elpasolite series, LnCl_6_^3−^. Since the Ln^3+^ ion is situated at a centrosymmetric site, pure and forced electric dipole allowed transitions are forbidden in the 4f^N^ – 4f^N^ optical spectra of hexanitritolanthanates. The electronic emission and absorption spectra comprise zero phonon lines enabled by the magnetic dipole mechanism together with sidebands of ungerade vibrations. Bünzli *et al*.^7^ made the first comprehensive study of the electronic spectra of these complexes at room and low temperatures, for Eu(NO_2_)_6_^3−^, and found the long lifetime of 10.9 ms for the ^5^D_0_ state at 4.2 K. Analysis of the quenching of emission at higher temperatures yielded the activation energy of 2250 cm^−1^. The quenching of ^5^D_0_ emission is unusual because the energy gap below this state is more than 11000 cm^−1^. A subsequent investigation focused upon the rich vibronic structure of the NO_2_^−^ ion in the Ln(NO_2_)_6_^3−^ series by recording the ultraviolet absorption spectra^8^. The lowest energy zero phonon line of NO_2_^−^ (at ~500 nm; ~20000 cm^−1^) is due to the spin-forbidden ^1^A_1_ → ^3^B_1_ (C_2v_) (S_0_ → T_1_) transition^9^. The first dipole allowed transition, S_0_ → S_1_: ^1^A_1_ → ^1^B_2_ of the NO_2_^−^ ion is at 384.9 nm in NaNO_2_^9^.

In view of the high coordination number of the lanthanide ion in the hexanitritolanthanate anion, we envisaged that the properties of the cerium complex would be of interest. First, we anticipated a large Stokes shift (Fig. S1) between the emission and absorption spectral bands. The Stokes shift could thus provide long wavelength cerium(III) emission. Second, the repercussions of this shift upon the temperature quenching of the emission spectrum would need to be studied. In order to investigate these processes, we synthesized Cs_2_NaCe(NO_2_)_6_ and determined the crystal structure and vibrational properties, as well as measuring the electronic spectra at temperatures down to 10 K. It was indeed demonstrated that the Stokes shift is the largest ever reported for Ce^3+^ emission in oxygen coordination.

## Methods

### Synthesis

The crystals of Cs_2_NaCe(NO_2_)_6_ were synthesized by dissolution of 337 mg (2 mmol) CsCl (Strem, 99.999%), 58 mg (1 mmol) NaCl (Dieckman, AR) and 373 mg (1 mmol) CeCl_3_.7H_2_O (Sigma-Aldrich, 99.9%) in 6 ml 37% aqueous HCl at 150 °C to obtain a white solid. A saturated solution of 1 ml NaNO_2_ (9 M) was then added and yellow precipitate was obtained. A transparent solution was obtained after adding 5 ml H_2_O. The solution was housed in a desiccator in a refrigerator at 4 °C. After two days, transparent, yellowish crystals were obtained which were removed from the mother liquor and dried. Powders were also prepared by precipitation according to the method of Roser and Corruccini^6^. Cs_2_NaLa(NO_2_)_6_ and Cs_2_NaY_0.96_Ce_0.04_(NO_2_)_6_ were prepared analogously.

### Instrumentation

**Figure 1:**
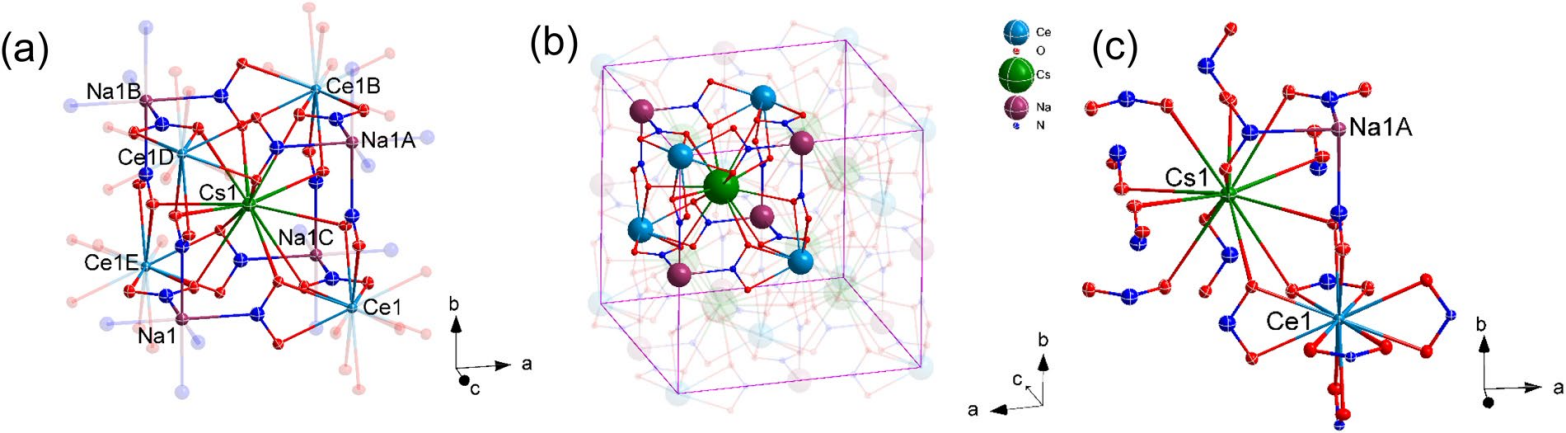
(**a**) A portion of the structure of Cs_2_NaCe(NO_2_)_6_. Symmetry code: A: 0.5 + *x*, 0.5 + *y*, *z*; B: *x*, 0.5 + *y*, −0.5 + *z*; C: 0.5 + *x*, *y*, −0.5 + *z*; D: −0.5 + *x*, 0.5 + *y*, *z*; E: −0.5 + *x*, *y*, −0.5 + *z*; (**b**) Diagram of the cubic Cs_2_NaCe(NO_2_)_6_ lattice. (**c**) Perspective view for the coordination environment of both Cs^+^ and Ce^3+^ cations in the lattice.

X-ray diffraction patterns of crystals were recorded with a Bruker AXS D8 Advance X-Ray Diffractometer using non-monochromated Cu K_α_ X-rays (λ = 1.5418 Å). FT-IR spectra were recorded at room temperature in the range from 400 to 4000 cm^−1^ using a PerkinElmer Paragon 1000 PC spectrometer with a resolution of 4 cm^−1^. Raman spectra were taken by a Perkin-Elmer Spectrum 2000 spectrometer using a resolution of 4 cm^−1^ at room temperature. The emission and excitation spectra were recorded by a Horiba Fluorolog-3 spectrophotometer using a 450 W xenon lamp as the continuous light source and the signal was detected by a Hamamatsu R928 photomultiplier. The crystal was cooled down by an Optical Cryostat-CS202I-DMX-1SS from Advanced Research Systems Instruments Inc. Luminescence lifetimes were measured by a 340 nm laser diode with 1 ns pulse width. The laser system consisted of an Nd:YAG pump laser, a third-order harmonic generator (THG at 355 nm, 120 mJ), and an optical parametric oscillator (OPO, Spectra-Physics versaScan and UVScan) with a pulse duration of 8 ns and repetition frequency of 10 Hz. X-ray photoelectron spectra (XPS) were recorded by a SKL-12 spectrometer modified with a VG CLAM 4 multichannel hemispherical analyser. Emission spectra were recorded using a Horiba 0.5 m monochromator (iHR550) equipped with a 600 groove mm^−1^ grating blazed at 800 nm and with a CCD detector (Syncerity, 300–1100 nm). Selected crystals were used for intensity data collection on a Bruker AXS Kappa Apex II Duo diffractometer at 173 K using frames of oscillation range 0.3°, with 2° < θ < 28°. An empirical absorption correction was applied using the SADABS program^10^. The structures were solved by the direct method and refined by full-matrix least-squares on *F*^2^ using the SHELXTL program package^11^, (Fig. 1).

## Calculations

### Ligand electronic spectra

**Figure 2:**
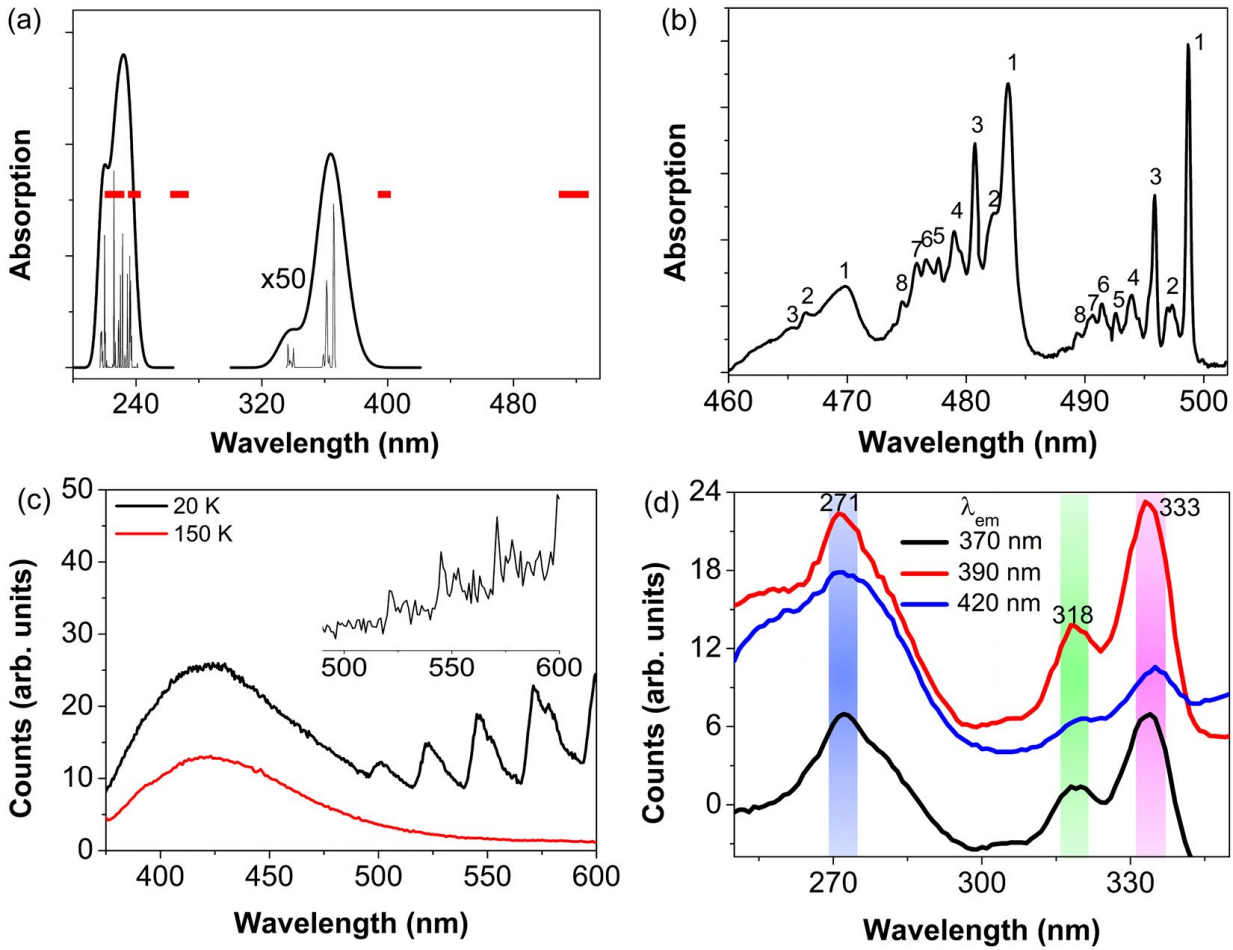
Spectra of hexanitrito anion. (**a**) Simulated absorption spectrum using Q(NO_2_)_6_^3−^ ion, where Q is a +3 Sparkle, in ORCA with the PBeh-3c functional. The inner spectra used FWHM of 50 cm^−1^ whereas the broadened spectrum used FWHM 1500 cm^−1^. Vibrational modes are omitted. The locations of triplet states are indicated in red. The Ce-O bond distances were optimized between 2.378–2.400 Å, mean 2.388 Å. (**b**) The single crystal triplet state absorption spectrum of Cs_2_NaCe(NO_2_)_6_ at 10 K; (**c**) Triplet state emission for Cs_2_NaLa(NO_2_)_6_ at 20 K and singlet state emission at 20 K and 150 K. The inset shows the triplet emission at higher resolution. (**d**) The excitation spectrum of singlet emission of Cs_2_NaLa(NO_2_)_6_ at 20 K.

The structure of Q(NO_2_)_6_^3−^ (where Q is a 3 + Sparkle) was optimized in ORCA^12,13^ using scalar relativistic and generalized gradient approximation (GGA) calculations. First, the BP86 functional was used with the basis set ZORA-def2-TZVP^14^ which is a relativistically recontracted version of the all-electron def2-TZVP Ahlrichs basis^15,16^. The SARC/J auxiliary basis set, which is a decontracted def2/J auxiliary was employed. A time dependent density functional theory (td-dft) calculation was then carried out. Alternatively, the composite approach PBEh-3c was employed with the basis def2-mSVP and the auxiliary basis def2/J^17^. This functional is a reparameterized version of PBE0 (with 42% HF exchange) that uses a double-zeta basis set, def2-mSVP (unlike the minimal basis set in HF-3c) and adds 3 corrections that correct for dispersion (via D3), basis set superposition (via gCP) and other basis set incompleteness effects. The results of the td-dft calculation were in closer agreement with experiment and are as shown in Fig. 2(a). The structure was drawn in Chemcraft^18^ according to the X-ray data and exported into ORCA^12,13^.

### Ab-initio calculation of Ce^3+^ crystal field parameters (CFP) and 5d^1^(1,2) splitting

We adopted a novel method for the 4 f^1^ electron CFP, which was developed by Novak and his co-workers^19^ to carry out first-principles calculations of the CFP of the 4 f configuration of a rare earth ion doped in a crystal host. Their calculated results match very well with the observed spectroscopic and magnetic data in lattices like aluminates, gallates, manganites and LaF_3_^19–22^. In fact, the method was based on Richter *et al*.’s^23^ earlier attempt to employ density functional theory (DFT) for band structure calculation of the lanthanide 4f-shell ions with transformation of the Kohn-Sham Hamiltonian to the Wannier basis^19–21^. For the present first-principles calculations of the CFP for the 5d^1^ electron of Ce^3+^, Novak’s novel method was also adopted with specific modifications and the whole procedures are concisely outlined as follows:

1. The WIEN2k program^24^ was applied for standard self-consistent band calculation (for solution of the Kohn-Sham equations of the DFT) with explicit incorporation of the 5d state in the core, giving rise to the crystal potential for subsequent calculations.
2. The oxygen ligand 2 s and 2p states and lanthanide 5d states were treated as valence states for the calculation of the effective crystal field Hamiltonian from ingredients involving the shape of the 5d orbital, the effective potential and the hybridization with the oxygen ligand orbitals in which the energy difference between the 5d and ligand states is taken as a ‘hybridization’ parameter.
3. The Wien2wannier^25^ and Wannier90^26^ computer packages were employed to transform the lanthanide 5d band states to the Wannier basis.
4. The local d-orbital Hamiltonian was extracted from the Wannier basis for subsequent extraction of the CFP.
5. The local d-orbital Hamiltonian was expanded in a series of spherical tensor operators to get values of the CFP for the 5d electron.

One of the present authors (Y. Y. Yeung) has developed a set of user-friendly subroutines in his *f-Spectra* package to carry out the last two steps for both d- and f-orbital electrons of any rare earth ion and to automatically set the required input parameters for the first two steps. It is noted that band-structure calculations should be performed with the Wien2k software package, which is based on the full-potential linearized augmented plane wave method and is often considered as the most accurate package for calculating the electronic properties of atoms with highly correlated electrons like 4 f and 5d electrons of rare earths. Other packages are not accurate enough for the present approach because the small energies for crystal field splittings are easily affected by minor inaccuracies in the calculations of band structures.

The atomic positions were taken from the cif file for Cs_2_NaCe(NO_2_)_6_ (and the same for other lanthanide ions). The oxygen-ligand distance employed was 2.6525 Å.

## Results and Discussion

### Crystal structure

Complex Cs_2_NaCe(NO_2_)_6_ crystallizes in a cubic space group (#202) with $\text{Fm}\bar{{\rm{3}}}$ lattice symmetry. The crystal data are summarized in Tables 1 and S1–S4. In this face-centered cubic structure, the central Cs^+^ ion is coordinated to twelve oxygen donor atoms from twelve symmetry-related nitrite ligands in the distance of 3.314(1) Å, as shown in Fig. 1. On the other hand, the Ce^3+^ ions, at two pairs of diagonal corners with an unusual tetrahedral *T*_*h*_ point group symmetry, are coordinated to twelve oxygen donor atoms from six symmetry-related nitrite groups. The Ce-O bond distance with twelve-coordinated oxygen is 2.653(1) Å, compared with the distance 2.82(2) Å reported for the corresponding La complex^27^. Six nitrogen atoms from the symmetrical nitrite groups are attached to each Na^+^ cation, located at four corners, to construct the face-centered cubic structure.

**Table 1:** Crystal data and structure refinement for Cs_2_NaCe(NO_2_)_6_.

Item	Parameter
Empirical formula	Cs_4_N_12_Na_2_O_24_Ce_2_
Formula weight	1409.98
Crystal system	Cubic
Space group	$\text{Fm}\bar{{\rm{3}}}$
a = b = c (Å)	11.1861(4)
α = β = γ (°)	90
Volume (Å^3^)	1399.70(15)
*Z*	2
ρ calc (g cm^−3^)	3.345
μ (mm^−1^)	8.483
*F* (000)	1268.0
Reflections collected	8471
Goodness of fit on *F*^2^	1.294
Final *R* indexes [*I* ≥ 2σ (*I*)]	*R*^1^ = 0.0139, *wR*^2^ = 0.0300
Final *R* indexes [all data]	*R*^1^ = 0.0143, *wR*^2^ = 0.0302

### Ligand electronic spectra

The previous assignments for the ultraviolet spectra of the NO_2_^−^ ion have agreed that there are three electric dipole allowed transitions from the ^1^A_1_ ground state to ^1^B_1_ (~360 nm, ε~28), ^1^A_2_ (~290 nm, ε~9) and ^1^B_2_ (~210 nm, ε~5000)^9^, where the wavelength values indicate the maxima of the absorption bands. The lowest energy transition, ^1^A_1_ → ^3^B_2_, is enhanced by about 100 times in Cs_2_NaLn(NO_2_)_6_ compared with NaNO_2_ due to increased spin-orbit coupling by the heavy atom effect^8^. The NO_2_^−^ transitions were investigated in Cs_2_NaLa(NO_2_)_6_ in addition to the Ce^3+^ analogue. Simulations of the NO_2_^−^ absorption spectra were carried out using the PBeh-3c functional^17^ in the ORCA^12,13^ program. The geometry optimization in ORCA did not give equal Ce-O bond distances. Hence each NO_2_^−^ level was split into 6 levels in this case. In *T*_*h*_ symmetry, with six NO_2_^−^ groups, the B_1_ and B_2_ levels only each split into 2 levels, T_g_ + T_u_, and the transition to the latter is electric dipole allowed from the A_g_ ground state. Figure 2(a) shows the result of the PBeh-3c calculation with broadened absorption bands due to S_0_ → S_1–3_ transitions at 363, 336 and 232 nm. The locations of triplet states are also indicated in the diagram, with the lowest one, S_0_ → T_1_, calculated to be at 525 nm.

The 10 K single crystal triplet state absorption spectrum of Cs_2_NaCe(NO_2_)_6_ is displayed in Fig. 2(b). The zero phonon line is the band at longest wavelength, observed at 499 nm (20053 cm^−1^), and a linear trend to higher energy is observed for this transition across the lanthanide series (Fig. S6). The peaks marked 1–8 in the figure exhibit a repeat with the interval corresponding to the totally symmetric NO_2_^−^ scissor frequency of 627 cm^−1^ in the T_1_ state and a marked broadening occurs at shorter wavelengths. Triplet state emission is observed at 20 K for Cs_2_NaLa(NO_2_)_6_, commencing to low energy of 495 nm, as shown in Fig. 2(c), with the major vibrational progression frequency of ~820 cm^−1^ corresponding to the totally symmetric NO_2_^−^ scissor mode in the S_0_ state. Excitation into either one of the NO_2_^−^ excited singlet states of Cs_2_NaLa(NO_2_)_6_ at 20 K gives a broad emission band with maximum at ~410 nm, accompanied by the triplet state emission (Fig. 2(c)). The band shifts to lower energy with increasing temperature and the triplet emission is quenched. The broad emission band was confirmed to be a transition between singlet states from the measured lifetime at wavelengths between 370–420 nm, <1 ns at 20 K (Fig. S7). The excitation spectrum of this singlet emission (Fig. 2(d)) shows singlet state absorption bands at 333, 318 and 271 nm.

### Cerium(III) electronic spectra

**Figure 3:**
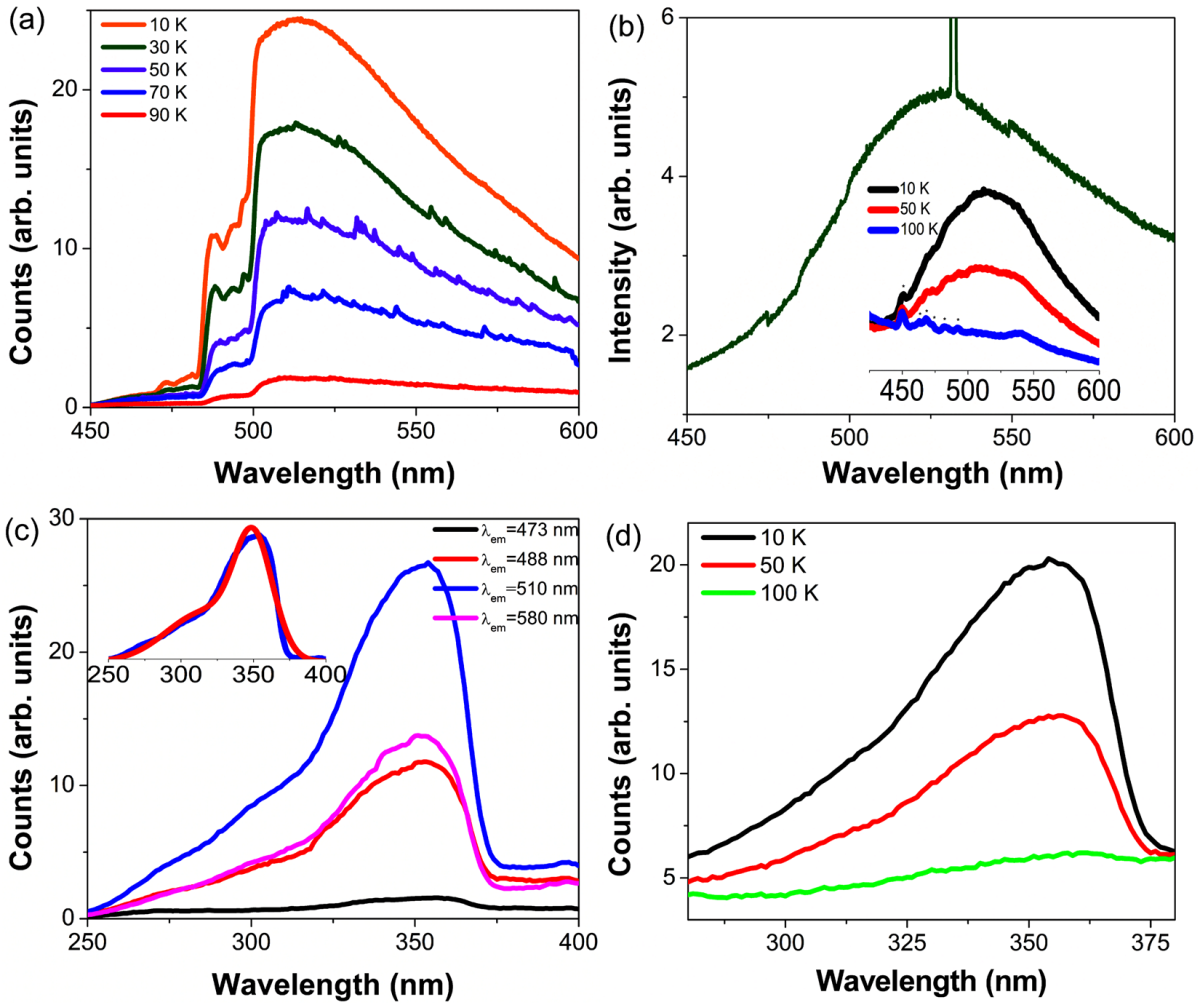
Emission (**a**), (**b**) and excitation (**c**), (**d**) spectra of Cs_2_NaCe(NO_2_)_6_. (**a**) 350 nm excited spectra between 10–90 K taken with the Fluorolog instrument; (**b**) 355 nm YAG:Nd^3+^ laser excited spectrum. The CCD windows exhibit slight shifts at 475 nm and 549 nm and the second harmonic of the laser gives the sharp line at 523 nm. The inset shows the temperature dependence of a further sample excited by 350 nm radiation from a xenon lamp, monitored by the Fluorolog instrument, with xenon lines starred. Excitation spectra of (**c**) using various emission wavelengths at 10 K and (**d**) of 510 nm emission at 10, 50 and 100 K. The inset of (**c**) shows the fitting in red of the spectrum monitoring 510 nm emission by two Gaussian peaks centered at 352 nm and 300 nm.

The 4f^1^ Ce^3+^ ion has a simple electronic energy structure, with the ^2^F_5/2_ ground state and the *J*-multiplet ^2^F_7/2_ at ~2000 cm^−1^ to higher energy. The 4f^1^ → 5d^1^ transition is located from the near red up to the ultraviolet spectral region^28–30^ depending upon the crystal field and nephelauxetic effect experienced by the Ce^3+^ ion. There are only two 5d Ce^3+^ crystal field levels, 5d^1^(1,2) in *T*_*h*_ symmetry Ce(NO_2_)_6_^3−^ and transitions to both are electric dipole allowed. The room temperature diffuse reflection spectrum of Cs_2_NaCe(NO_2_)_6_ (Fig. S8) exhibits a weak NO_2_^−^ triplet state absorption starting at ~500 nm, with a stronger absorption band to shorter wavelength comprising the Ce^3+^ 4f^1^ → 5d^1^ and NO_2_^−^ singlet state transitions. A broad band with maximum intensity near 514–527 nm is observed in the low temperature emission spectrum of this compound which we assign to the Ce^3+^ 5d^1^ → 4f^1 2^F_5/2,7/2_ transitions. The emission band shape changes for different crystals due to varying self-absorption by the NO_2_^−^ triplet state, having a structured appearance to shorter wavelength of 500 nm. This is evident in Fig. 3(a) but not so in Fig. 3(b) for a different crystal. At 10 K, the emission is not excited by wavelengths longer than 380 nm (Fig. S9). At 20 K, the Ce^3+^ 5d^1^ lifetime was monitored at various wavelengths from 450 nm to 525 nm, being monoexponential and giving the average value of 25.8 ± 1.3 ns (Fig. S10), similar to that in YAG:Ce^3+^^31^.

The low temperature excitation spectra of the Ce^3+^ emission at various wavelengths are displayed in Figs 3(c) and S11. Notice that the broad excitation band is associated with emission from Ce^3+^ and not NO_2_^−^ (Fig. S12) and that the excitation peak maximum varies slightly from sample to sample. The excitation band may be fit by two Gaussians with maxima at 352 and 300 nm, as in the inset, Fig. 3(c). These two wavelengths are associated with vibronic maxima of the transitions 4f^1 2^F_5/2_ → 5d^1^(1,2) so that the 5d^1^ splitting is 4925 cm^−1^. The *first principles* calculation using the crystal structure of Cs_2_NaCe(NO_2_)_6_ gives the 5d^1^(1,2) splitting as 4625 cm^−1^ (Refer to the SI, Table S6), which is in reasonable agreement.

### Temperature dependence

The dramatic quenching of emission with temperature (*T*_*q*_, the temperature where emission intensity is reduced to one-half of the initial value = 53 ± 2 K) is illustrated in Fig. 3(a) and the inset in 3(b). The Ce^3+^ emission is almost quenched at 100 K (Fig. S13). A corresponding quenching is observed in the excitation spectra, Fig. 3(d).

**Figure 4:**
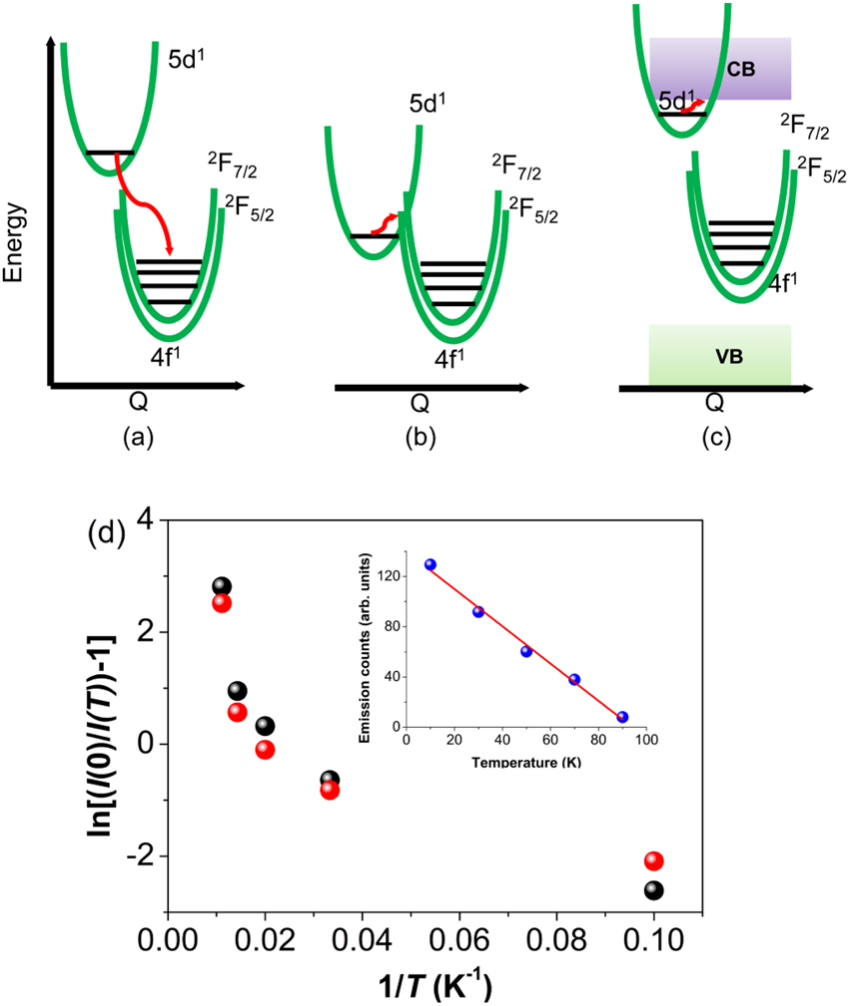
(**a**–**c**) Mechanisms of thermal quenching of Ce^3+^ 5d^1^ → 4f^1^ emission. (**d**) Plot relating a function of integrated counts, *I*(*T*), of Cs_2_NaCe(NO_2_)_6_ emission from 480–600 nm under 350 nm excitation (black sphere) and integrated counts from excitation spectrum between 250–380 nm when monitoring 510 nm emission (red sphere) versus reciprocal temperature (with *T* from 10 K to 90 K) for Cs_2_NaCe(NO_2_)_6_. The values of *I*(0) were determined from extrapolation of the linear plots of *I*(*T*) versus *T* (for example: inset, *I*(0) from emission).

Although the maximum phonon energy in the crystal is quite high (1334 cm^−1^), the quenching mechanism of 5d^1^ → 4f^1^ emission by multiphonon relaxation is discounted because the gap from 5d^1^ to 4f^1^ is bridged by ~16 phonons. By contrast, the 4f^2^ → 4f^2^ emission from the ^3^P_0_ level of Pr^3+^ in Cs_2_NaPr(NO_2_)_6_ is quenched^3^ because the energy gap ^3^P_0_ - ^1^D_2_ is spanned by only 3 phonons. Three alternative quenching mechanisms are depicted in Fig. 4(a–c) by reference to configuration coordinate and valence band (VB)/conduction band (CB) diagrams. There have been various points of view put forward for other Ce^3+^ systems, notably YAG:Ce^3+^^32^, with respect to these mechanisms. The energy transfer to another species is considered unlikely since no emission is observed to lower energy. The temperature-induced crossover to another potential energy surface (Fig. 4(b)) and photoionization to the CB (Fig. 4(c)) are candidates.

An Arrhenius plot of ln{[*I*(0)/*I*(*T*)]−1} (where *I*(*T*) denotes the counts at temperature *T*) against reciprocal temperature may be employed to estimate the activation energy of the quenching process. In the present case, the use of emission counts or excitation counts for several different samples gives linear plots of intensity versus temperature in the range from 20–100 K (e.g. inset Fig. 4(d)). The corresponding plots of ln{[*I*(0)/*I*(*T*)]−1} versus 1/*T* are not linear (Fig. 4(d)) and show that the activation energy *E*_*a*_ for the quenching process changes from 29 ± 7 cm^−1^ in the range from 10–30 K to 429 ± 200 cm^−1^ in the range from 70–90 K. The overall quenching behaviour from 20–250 K is shown in Fig. S14 and follows an exponential decrease in intensity.

### Stokes shift

**Figure 5:**
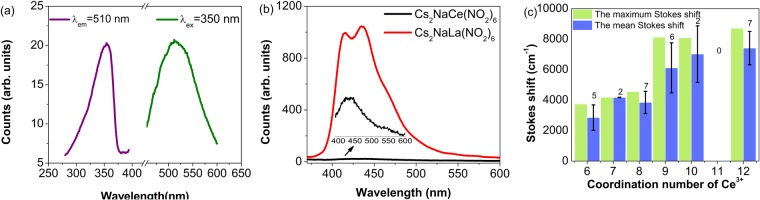
(**a**) Large Stokes shift of Ce^3+^ in Cs_2_NaCe(NO_2_)_6_; (**b**) Room temperature 350 nm-excited singlet emission in Cs_2_NaLa(NO_2_)_6_ (red) and Cs_2_NaCe(NO_2_)_6_ (black). The scale expansion is shown for the latter. (**c**) Stokes shifts for different coordination numbers of Ce-O systems. The number of compounds of each type is indicated together with the standard deviation. Data from Table S7 and this work.

The peak maxima are at 355 nm (excitation spectrum) and 514 nm (emission spectrum) in Fig. 5(a) for Cs_2_NaCe(NO_2_)_6_ so that the Stokes shift of 8715 cm^−1^ is the maximum reported for cerium coordinated to oxygen in the literature. The progression-forming mode is most likely the totally-symmetric Ce-O stretch, which has a magnitude near 240 cm^−1^ (Fig. S5(a)). Technically, this Stokes shift does not correspond to the ‘same transition’ in absorption (4 f^1 2^F_5/2_ → 5d^1^(1,2)) and emission (5d^1^(1) → 4f^1 2^F_5/2_,_7/2_)^33^. The shift represents the interactions taking place within the CeO_12_ cluster, although the embedding effect of this cluster is also important. Contrary to the traditional depiction in many textbooks, the excitation of the Ce^3+^ 4f electron to the first 5d state often involves a bond length contraction, not expansion^34,35^. In fact, in solution, this contraction can lead to the expulsion of a ligand^36^. The great change in equilibrium bond distance between the ground and excited states gives the possibility that the potential energy curves overlap (Fig. 4(b))^37^. Hence inefficient fluorescence occurs. The location of the 4f^1^ → 5d^1^(1) zero phonon line can be roughly estimated from the midway point of the band maxima or band onsets in Fig. 5(a) and is in the region of 400–420 nm. The thermal crossover thus occurs just above this energy (i.e. above 23800–25000 cm^−1^) because vibrational relaxation to the electronic ground state is much faster than the crossover rate. Hence the crossover, Fig. 4(b), could occur to 4f^1^ Ce^3+^ or to possibly to the NO_2_^−^ singlet state potential energy curve. In fact, just as for Cs_2_NaLa(NO_2_)_6_, room temperature singlet state emission is also observed for Cs_2_NaCe(NO_2_)_6_ (Fig. 5(b)). The change in activation energy with temperature arises from the bond length change and relative zero phonon line shift with temperature, which serve to displace the two potential energy curves.

Literature tabulations of Stokes shifts^38–41^ include materials with Ce^3+^ ions occupying several sites in the crystal lattice, such as the hosts Lu_4_AI_2_O_9_, LaLuO_3_, Ba_3_Gd(BO_3_)_3_ and Mg_2_Y_8_(SiO_4_)_6_O_2_. We have excluded these more complex materials, which may not be sufficiently-well characterized, from Table S7 which compares the Stokes shift for cerium in oxygen coordination with other parameters. The plot of Stokes shift versus coordination number, Fig. 5(c), demonstrates larger Stokes shifts for Ce^3+^ ions with higher coordination number showing that Ce^3+^ ions with larger ionic radii (i.e. from Ce^3+^(VI) 101 pm to Ce^3+^(XII) 134 pm) can contract more when transiting from 4 f to 5d. Plots of Stokes shift against mean or minimum Ce-O distance indicate that other factors, such as formal charge, vibrational frequency, thermal population of excited states and change in excited state geometry also play a role (Fig. S15 and Table S7). Small Stokes shifts have been associated with rigid lattices^42^. The weaker metal-ligand bonding is in line with a lower value of *T*_*q*_.

## Conclusions

In summary, the hexanitrito complex Cs_2_NaCe(NO_2_)_6_, with 12-coordinated Ce^3+^ situated at a site of *T*_*h*_ symmetry, exhibits a very large energy shift between the peak maxima in absorption and emission. The 4f^1^ ground state and 5d^1^ potential surfaces are displaced so much along the configuration coordinate that overlap takes place above the 5d^1^ minimum, leading to thermal quenching of emission at a low temperature. The features due to NO_2_^−^ and Ce^3+^ ions in the electronic spectra have been rationalized by theory.

## Electronic supplementary material

Supplementary Information

## References

[1] Clack DW, Reinen D (1980). Molecular orbital calculations on complexes with strongly Jahn-Teller unstable transition metals: The hexanitro-copper(II) and cobalt(II) ions. Solid State Commun..

[2] Barnes, J. C. & Peacock, R. D. Hexanitrolanthanates(III). *J. Chem. Soc. A*. 558–562 (1971).

[3] Li WY, Ning LX, Faucher MD, Tanner PA (2011). Experimental and theoretical studies of the vibrational and electronic spectra of a lanthanide ion at a site of *T*_*h*_ symmetry: Pr^3+^ in Cs_2_NaPr(NO_2_)_6_. Inorg. Chem..

[4] Tanner PA, Li WY, Ning LX (2012). Electronic spectra and crystal field analysis of Tb^3+^ in Cs_2_NaTb(NO_2_)_6_: Tb^3+^ situated at a site of *T*_*h*_ symmetry. J. Phys. Chem. C..

[5] Tanner PA, Li WY, Ning LX (2012). Electronic spectra and crystal-field analysis of europium in hexanitritolanthanate systems. Inorg. Chem..

[6] Roser MR, Corruccini LR (1990). Magnetic susceptibilities of rare-earth ions in an unusual tetrahedral site. Phys. Rev. B..

[7] Bűnzli JCG, Petoud S, Moret E (1999). Luminescent properties of the hexakis(nitrito)europate(III) ion [Eu(NO_2_)_6_]^3−^. Spectrosc. Lett..

[8] Kirschner AV (1998). Spectroscopy of hexanitritoelpasolite crystals: the effect of the rare-earth ion on the progressions in the nitrite vibration. Spectrochim. Acta. A..

[9] Sidman JW (1957). Electronic and vibrational states of the nitrite ion. J. Am. Chem. Soc..

[10] Sheldrick, G. M. SADABS: Program for empirical absorption correction of area detector data (University of Göttingen: Göttingen, Germany, 1996).

[11] Sheldrick, G. M. SHELXTL 5.10 for windows structure determination software programs (Bruker Analytical X-ray Systems, Inc., Madison, Wisconsin, USA, 1997).

[12] Neese F (2012). The ORCA program system. WIREs Comput. Mol. Sci..

[13] Neese F (2018). Software update: the ORCA program system, version 4.0. WIREs Comput. Mol. Sci..

[14] Weigend F, Ahlrichs R (2005). Balanced basis sets of split valence, triple zeta valence and quadruple zeta valence quality for H to Rn: design and assessment of accuracy. Phys. Chem. Chem. Phys..

[15] Weigend F (2006). Accurate Coulomb-fitting basis sets for H to Rn. Phys. Chem. Chem. Phys..

[16] Pantazis DA, Neese F (2012). All-electron scalar relativistic basis sets for the 6p elements. Theor. Chem. Acc..

[17] Grimme S, Brandenburg JG, Bannwarth C, Hansen A (2015). Consistent structures and interactions by density functional theory with small atomic orbital basis sets. J. Chem. Phys..

[18] Chemcraft. Available at, https://www.chemcraftprog.com/.

[19] Novák P, Knĩžek K, Kuneš J (2013). Crystal field parameters with Wannier functions: Application to rare-earth aluminates. Phys. Rev. B..

[20] Novák P, Knĩžek K, Maryško M, Jirák Z, Kuneš J (2013). Crystal field and magnetism of Pr^3+^ and Nd^3+^ ions in orthorhombic perovskites. J. Phys: Condens. Matter..

[21] Novák P, Nekvasil V, Knĩžek K (2014). Crystal field and magnetism with Wannier functions: orthorhombic rare-earth manganites. J. Magn. Magn. Mater..

[22] Novák P, Kuneš J, Knĩžek K (2014). Crystal field of rare earth impurities in LaF_3_. Opt. Mater..

[23] Richter M, Oppeneer PM, Eschrig H, Johansson B (1992). Calculated crystal-field parameters of SmCo_5_. Phys. Rev. B..

[24] Blaha, P., Schwarz, K., Madsen, G., Kvasnicka, D. & Luitz, J. WIEN2K: An augmented plane wave and local orbitals program for calculating crystal properties (Vienna University of Technology: Austria, 2001).

[25] Kuneš J (2010). Wien2wannier: From linearized augmented plane waves to maximally localized Wannier functions. Comput. Phys. Commun..

[26] Mostofi AA (2008). Wannier90: a tool for obtaining maximally-localised wannier functions. Comput. Phys. Commun..

[27] Barnes JC, Al-Rasoul K, Harkins P (1980). The crystal structure of lanthanoid hexanitrite complexes. J. Chem. Soc. Pak..

[28] Xia ZG, Meijerink A (2017). Ce^3+^-Doped garnet phosphors: composition modification, luminescence properties and applications. Chem. Soc. Rev..

[29] Ueda J, Tanabe S, Nakanishi T (2011). Analysis of Ce^3+^ luminescence quenching in solid solutions between Y_3_Al_5_O_12_ and Y_3_Ga_5_O_12_ by temperature dependence of photoconductivity measurement. J. Appl. Phys..

[30] Gektin A (2009). Luminescence of heavily Ce-doped alkaline-earth fluorides. J. Lumin..

[31] He XW (2016). Effects of local structure of Ce^3+^ ions on luminescent properties of Y_3_Al_5_O_12_: Ce nanoparticles. Scientific Reports..

[32] Ivanovskikh KV, Ogiegło JM, Zych A, Ronda CR, Meijerink A (2013). Luminescence temperature quenching for Ce^3+^ and Pr^3+^*d-f* emission in YAG and LuAG. ECS J. Solid. State. Sci. Technol..

[33] Tanner PA (2013). Some misconceptions concerning the electronic spectra of tri-positive europium and cerium. Chem. Soc. Rev..

[34] Barandiarán Z, Seijo L (2006). On the bond length change upon 4*f*^1^ → 5*d*^1^ excitations in eightfold coordination: CaF_2_:Ce^3+^ cubic defects. Theor. Chem. Acc..

[35] Barandiarán Z, Seijo L (2003). Quantum chemical analysis of the bond lengths in *f*^*n*^ and *f*^n−1^*d*^1^ states of Ce^3+^, Pr^3+^, Pa^4+^, and U^4+^ defects in chloride hosts. J. Chem. Phys..

[36] Wang JW, Mei Y, Tanner PA (2014). Luminescence properties, centroid shift and energy transfer of Ce^3+^ in aqueous chloride solutions. J. Lumin..

[37] Blasse G, Bril A (1968). Photoluminescent efficiency of phosphors with electronic transitions in localized centers. J. Electrochem. Soc..

[38] Dorenbos P, Andriessen J, Marsman M, van Eijk CWE (2001). On the Stokes shift of the Ce^3+^ 5d4f luminescence in inorganic crystals. Rad. Eff. Def. Solids..

[39] de Vries AJ, Blasse G (1986). On the possibility to sensitize Gd^3+^ luminescence by the Pr^3+^ ion. Mat. Res. Bull..

[40] Dorenbos P (2000). The 5d level positions of the trivalent lanthanides in inorganic compounds. J. Lumin..

[41] Blasse G, Bril A (1967). Investigation of some Ce^3+^-activated phosphors. J. Chem. Phys..

[42] van Krevel JWH, Hintzen HT, Metselaar R, Meijerink A (1998). Long wavelength Ce emission in Y–Si–O–N materials. J. Alloys Compds..

