# Supplementary material for: Massive Stokes shift in 12-coordinate Ce(NO2)63−: crystal structure, vibrational and electronic spectra
Source: Sci Rep. 2018 Nov 8;8:16557. doi: 10.1038/s41598-018-34889-4 (PMC6224556; doi:10.1038/s41598-018-34889-4)
Supplement: Supplementary file 1 — Supplementary Information [file 41598_2018_34889_MOESM1_ESM.pdf]

Supporting Information for

Massive Stokes shift in 12-coordinate $Ce(NO_2)_6^{3-}$: crystal structure, vibrational and electronic spectra

Yuxia Luo[a], Chun-Kit Hau[a], Yau Yuen Yeung[b], Ka-Leung Wong[a],* Kwok Keung Shiu[a], and Peter A. Tanner[a]*

[a]Department of Chemistry, Hong Kong Baptist University, 224 Waterloo Road, Kowloon, Hong Kong S.A.R., P.R. China

[b]Department of Science and Environmental Studies, The Education University of Hong Kong, 10 Lo Ping Road, Tai Po, New Territories, Hong Kong, P. R. China

Correspondence and requests for materials should be addressed to P.A.T. and K.-L.W. (email: peter.a.tanner@gmail.com klwong@hkbu.edu.hk)

Abstract: The Ce^{3+} ion in $Cs_2NaCe(NO_2)_6$ (**I**), which comprises the unusual T_h site symmetry of the $Ce(NO_2)_6^{3-}$ ion, demonstrates the largest Ce-O Stokes shift of 8715 cm^{-1} and the low emission quenching temperature of 53 K. The activation energy for quenching changes with temperature, attributed to relative shifts of the two potential energy curves involved. The splitting of the Ce^{3+} $5d^1$ state into two levels separated by 4925 cm^{-1} is accounted for by a first principles calculation using the crystal structure data of **I**. The NO_2^- energy levels and spectra were investigated also in $Cs_2NaLa(NO_2)_6$ and modelled by hybrid DFT. The vibrational and electronic spectral properties have been thoroughly investigated and rationalized at temperatures down to 10 K. A comparison of Stokes shifts with other Ce-O systems emphasizes the dependence upon the coordination number of Ce^{3+}.

Table of Contents

Figure. S1. Representation of the Stokes shift. 3

Figure. S2. Crystals of $Cs_2NaCe(NO_2)_6$ 3

Figure. S3. Room temperature X-ray diffractograms 4

Figure. S4. X-ray photoelectron spectra 4

Table. S1. Fractional atomic coordinates and equivalent isotropic displacement parameters 4

Table. S2. Anisotropic Displacement Parameters 4

Table. S3. Bond Lengths 5

Table. S4. Bond Angles 5

Figure. S5. (a) Raman and (b) FT-IR spectra of $Cs_2NaCe(NO_2)_6$ at room temperature.

(c) Plot of Raman vibrational frequencies for the series $Cs_2NaLn(NO_2)_6$. 6

Table. S5. Assignments for vibrational spectra of $Cs_2NaCe(NO_2)_6$ at 295 K. 6

Figure. S6. Trend in zero phonon line energy for $Cs_2NaLn(NO_2)_6$ series. 7

Table. S6. Ab initio crystal field parameters for Ln^{3+} in $Cs_2NaLn(NO_2)_6$ and 5d (1,2) crystal field splitting of Ce^{3+}. 7

Figure. S7. Measurements at various wavelengths of $S_1 \rightarrow S_0$ singlet emission lifetime of $Cs_2NaLa(NO_2)_6$ at 20 K. 7

Figure. S8. Room temperature diffuse reflection spectrum of $Cs_2NaCe(NO_2)_6$. 8

Figure. S9. 10 K emission spectra of $Cs_2NaCe(NO_2)_6$ using various excitation wavelengths. 8

Figure. S10. Measured Ce^{3+} lifetime at 20 K for various emission wavelengths of $Cs_2NaCe(NO_2)_6$. 8

Figure. S11. 10 K excitation spectrum of $Cs_2NaCe(NO_2)_6$ monitoring two emission wavelengths. 8

Figure. S12. 20 K excitation spectra of $Cs_2NaCe(NO_2)_6$ monitoring NO_2^- emission

at 420 nm (green) and Ce^{3+} emission at 546 nm (blue) and 571 nm (cyan). 9

Figure. S13. 100 K excitation and emission spectra of $Cs_2NaCe(NO_2)_6$. 9

Figure. S14. (a) Integrated 5d – 4f emission spectra of Ce^{3+} in $Cs_2NaCe(NO_2)_6$ under 333 nm excitation at different temperatures;

(b) Emission spectra between 490-560 nm for $Cs_2NaCe(NO_2)_6$ under 333 nm excitation from 150 K to 300 K;

(c) Arrhenius plot for the temperature range from 150-250 K. 9

References 10

Figure. S15 Plot of Stokes shift for Ce-O systems against (a) average Ce-O distance

and (b) shortest Ce-O distance from literature data, Table S7 and this work. 11

Table. S7. Stokes shifts and physical parameters of cerium-oxygen systems. 11

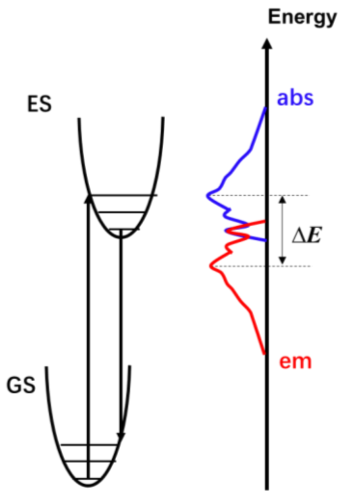

Figure. S1. Representation of Stokes shift. The Stokes shift (ΔE) occurs since electronic absorption from the electronic ground state takes place to excited vibrational states so quickly that nuclear movement is negligible. Then nonradiative relaxation (i.e. energy loss) occurs in the metastable excited state to the lowest vibrational levels from which emission occurs. The emission transition occurs without nuclear movement so that the terminal state is an excited vibrational level of the electronic ground state.

The synthesis of the compound

The main raw materials and their purity:

Reagent	Chemical formula	Purity	Formula Weight	Supplier
**lanthanum(III) chloride heptahydrate**	$LaCl_3.7H_2O$	99.999%	371.37	Sigma-Aldrich
**cerium(III) chloride heptahydrate**	$CeCl_3 \cdot 7H_2O$	99.9%	372.58	Sigma-Aldrich
**cerium(III) chloride heptahydrate**	$CeCl_3 \cdot 7H_2O$	99.999%	372.58	Sigma-Aldrich
**sodium chloride**	NaCl	AR	58.44	Dickman
**cesium chloride**	CsCl	99.999%	168.36	Strem
**sodium nitrite**	$NaNO_2$	AR	69	Dickman

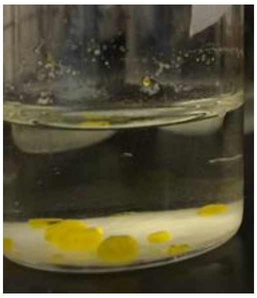

Figure. S2. Crystals of $Cs_2NaCe(NO_2)_6$ of dimension 1-3 mm.

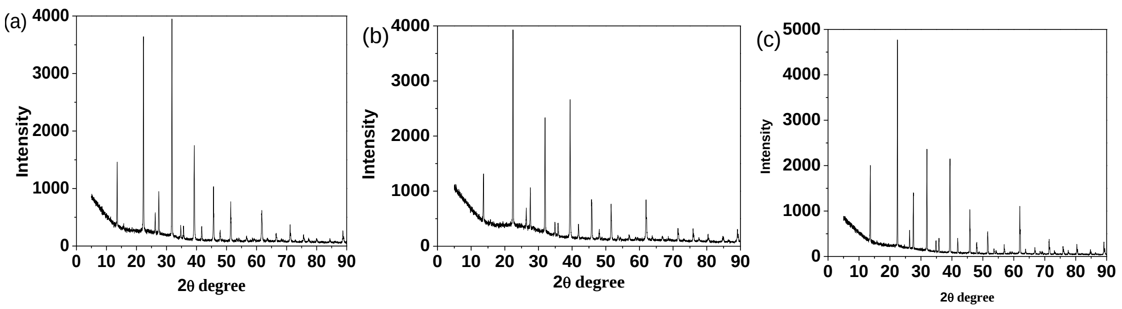

Figure. S3. Room temperature X-ray diffractograms of (a) $Cs_2NaLa(NO_2)_6$; (b) $Cs_2NaCe(NO_2)_6$ prepared from the $Cs_2NaCeCl_6$ elpasolite[1] and (c) $Cs_2NaCe(NO_2)_6$ prepared by the method of Roser and Coruccini[2] show that the two methods of synthesis give the same product and that $Cs_2NaLa(NO_2)_6$ and $Cs_2NaCe(NO_2)_6$ are isostructural.

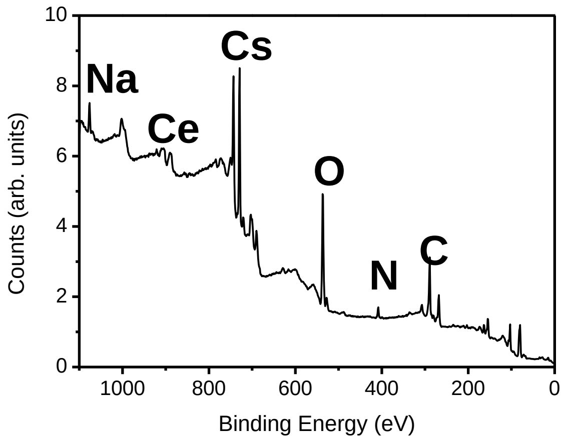

Figure. S4. The X-ray photoelectron spectrum shows the target elements and also the presence of Ce^{4+} impurity in $Cs_2NaCe(NO_2)_6$.

Crystal structure data of $Cs_2NaCe(NO_2)_6$.

Table. S1. Fractional atomic coordinates (×10[4]) and equivalent isotropic displacement parameters (Å^2×10^3). U(eq) is defined as 1/3 of of the trace of the orthogonalised U_{IJ} tensor.

Atom	*x*	*y*	*z*	U(eq)
Ce1	0	-5000	-5000	12.64(14)
O1	2175.5(13)	-5943.4(14)	-5000	27.1(3)
N1	2789(2)	-5000	-5000	24.8(5)
Cs1	2500	-7500	-7500	23.67(14)
Na1	5000	-5000	-5000	18.9(6)

Table. S2. Anisotropic Displacement Parameters (Å^2×10^3). The Anisotropic displacement factor exponent takes the form: $-2\pi^2[h^2a^{*2}U_{11}+2hka^*b^*U_{12}+\ldots]$.

Atom	U_{11}	U_{22}	U_{33}	U_{23}	U_{13}	U_{12}
Ce1	12.64(14)	12.64(14)	12.64(14)	0	0	0
O1	22.6(8)	24.8(8)	34.0(8)	0	0	2.2(6)
N1	16.3(11)	33.4(14)	24.6(12)	0	0	0
Cs1	23.67(14)	23.67(14)	23.67(14)	0	0	0
Na1	18.9(6)	18.9(6)	18.9(6)	0	0	0

Table. S3. Bond Lengths

Atom	Atom	Length (Å)	Atom	Atom	Length (Å)
Ce1	O1	2.6525(15)	Ce1	O1[7]	2.6525(15)
Ce1	O1[1]	2.6525(15)	Ce1	O1[8]	2.6525(15)
Ce1	O1[2]	2.6525(15)	Ce1	O1[9]	2.6525(15)
Ce1	O1[3]	2.6525(15)	Ce1	O1[10]	2.6525(15)
Ce1	O1[4]	2.6525(15)	Ce1	O1[11]	2.6525(15)
Ce1	O1[5]	2.6525(15)	O1	N1	1.259(2)
Ce1	O1[6]	2.6525(15)	N1	O1[2]	1.259(2)

[1]-X,+Y,+Z; [2]+X,-1-Y,-1-Z; [3]1/2+Y,+Z,-1/2+X; [4]-1/2-Z,-1/2-X,+Y; [5]-X,-1-Y,-1-Z; [6]-1/2-Y,-1-Z,-1/2-X; [7]-1/2-Y,+Z,-1/2+X; [8]1/2+Z,-1/2+X,-1-Y; [9]1/2+Z,-1/2+X,+Y; [10]1/2+Y,-1-Z,-1/2-X; [11]-1/2-Z,-1/2-X,-1-Y

Table. S4. Bond Angles

Atom	Atom	Atom	Angle (˚)	Atom	Atom	Atom	Angle (˚)
O1	Ce1	O1	68.59(2)	O1[10]	Ce1	O1[11]	133.12(7)
O1[2]	Ce1	O1[3]	133.12(7)	O1[10]	Ce1	O1[9]	68.59(2)
O1[4]	Ce1	O1[5]	133.12(7)	O1[9]	Ce1	O1[11]	68.59(2)
O1[6]	Ce1	O1[7]	68.59(2)	O1[4]	Ce1	O1[11]	111.41(2)
O1[2]	Ce1	O1[8]	180.0	O1[8]	Ce1	O1[11]	111.41(2)
O1[9]	Ce1	O1[6]	111.41(2)	O1[4]	Ce1	O1[9]	180.00(6)
O1	Ce1	O1[3]	68.59(2)	O1[3]	Ce1	O1[11]	68.59(2)
O1	Ce1	O1[10]	111.41(2)	O1[5]	Ce1	O1[11]	68.59(2)
O1[4]	Ce1	O1[8]	111.41(2)	O1[1]	Ce1	O1[6]	133.12(7)
O1[2]	Ce1	O1[11]	68.59(2)	O1	Ce1	O1[2]	111.41(2)
O1[1]	Ce1	O1[5]	111.41(2)	O1[4]	Ce1	O1[6]	68.59(2)
O1[1]	Ce1	O1[10]	180.0	O1[10]	Ce1	O1[6]	46.88(7)
O1[2]	Ce1	O1[5]	68.59(2)	O1[2]	Ce1	O1[6]	111.41(2)
O1[10]	Ce1	O1[7]	68.59(2)	O1[1]	Ce1	O1[2]	68.59(2)
O1[10]	Ce1	O1[3]	111.41(2)	O1[5]	Ce1	O1[6]	111.41(2)
O1	Ce1	O1[4]	46.89(7)	O1[8]	Ce1	O1[6]	68.59(2)
O1[10]	Ce1	O1[8]	68.59(2)	O1[11]	Ce1	O1[6]	180.0
O1[1]	Ce1	O1[11]	46.88(7)	O1[10]	Ce1	O1[2]	111.41(2)
O1[9]	Ce1	O1[8]	68.59(2)	O1[1]	Ce1	O1[7]	111.41(2)
O1[1]	Ce1	O1[4]	68.59(2)	O1	Ce1	O1[7]	111.41(2)
O1	Ce1	O1[5]	180.0	O1[4]	Ce1	O1[7]	68.59(2)
O1	Ce1	O1[6]	68.59(2)	O1[4]	Ce1	O1[2]	68.59(2)
O1[10]	Ce1	O1[5]	68.59(2)	O1[2]	Ce1	O1[7]	46.88(7)
O1[10]	Ce1	O1[4]	111.41(2)	O1[9]	Ce1	O1[7]	111.41(2)
O1[9]	Ce1	O1[5]	46.88(7)	O1[5]	Ce1	O1[7]	68.59(2)
O1[3]	Ce1	O1[6]	111.41(2)	O1[9]	Ce1	O1[2]	111.41(2)
O1[8]	Ce1	O1[5]	111.41(2)	O1[11]	Ce1	O1[7]	111.41(2)
O1	Ce1	O1[9]	133.11(7)	O1[3]	Ce1	O1[7]	180.0
O1[1]	Ce1	O1[3]	68.59(2)	O1[9]	Ce1	O1[3]	68.59(2)
O1[8]	Ce1	O1[7]	133.12(7)	O1	Ce1	O1[8]	68.59(2)
O1[4]	Ce1	O1[3]	111.41(2)	O1[8]	Ce1	O1[3]	46.88(7)
O1[1]	Ce1	O1[9]	111.41(2)	O1[1]	Ce1	O1[8]	111.41(2)
O1	Ce1	O1[11]	111.41(2)	N1	O1	Ce1	99.57(13)
O1[5]	Ce1	O1[3]	111.41(2)	O1[4]	N1	O1	114.0(2)

[1]-1/2-Y,+Z,-1/2+X; [2]1/2+Z,-1/2+X,-1-Y; [3]-1/2-Z,-1/2-X,-1-Y; [4]+X,-1-Y,-1-Z; [5]-X,-1-Y,-1-Z; [6]-1/2-Y,-1-Z,-1/2-X; [7]1/2+Z,-1/2+X,+Y; [8]-1/2-Z,-1/2-X,+Y; [9]-X,+Y,+Z; [10]1/2+Y,-1-Z,-1/2-X; [11]1/2+Y,+Z,-1/2+X

Vibrational spectra

The target compound $Cs_2NaCe(NO_2)_6$ was also characterized by vibrational spectroscopy (Fig. S5(a), (b)). The crystal has 66 modes of vibration, with 51 modes of the $Ce(NO_2)_6^{3-}$ moiety, many of which are degenerate. Following the previous elucidation of the normal modes, the assignments for the room temperature vibrational spectra are listed in Table S5. The most intense bands in the Raman and IR spectra correspond to N-O stretching modes. The assignments are firm for bands above 800 cm^{-1}. Fig. S5(c) compares the vibrational energies of the Raman bands with those available for other $Cs_2NaLn(NO_2)_6$ systems. Calculations have previously been presented for $Cs_2NaLn(NO_2)_6$ Ln = La, Pr.[3,4] Some reassignments have been made in Fig. S5(c), where clear trends are visible across the lanthanide series, as illustrated by the linear fittings. There is an increase in all vibrational energies (often very small) across the series as the ionic radius of the cation decreases, with the most striking trend being for the T_g NO_2^- wag vibration.

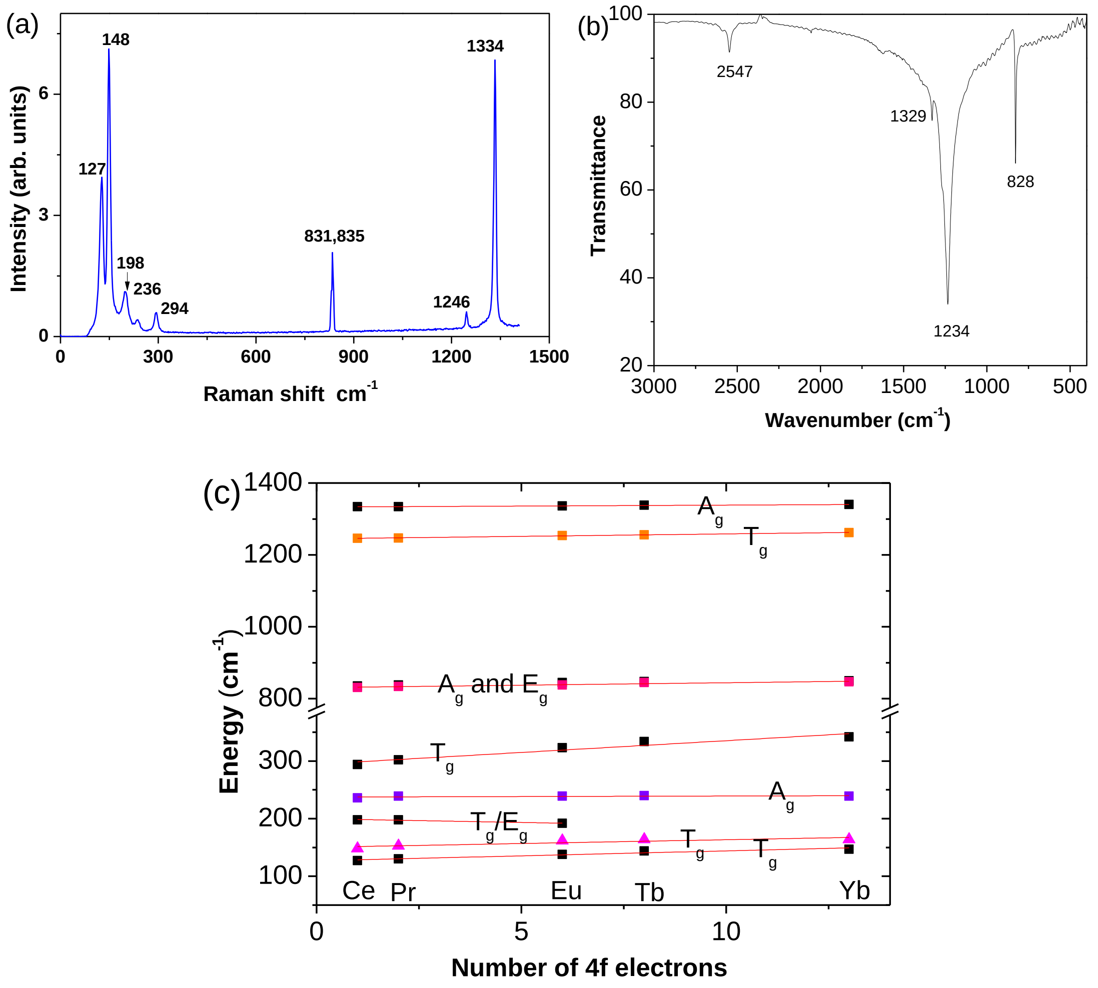

Figure. S5. (a) Raman and (b) FT-IR spectra of $Cs_2NaCe(NO_2)_6$ at room temperature. (c) Plot of Raman vibrational frequencies for the series $Cs_2NaLn(NO_2)_6$. Notice the ordinate scale break in (c).

Table. S5. Assignments for vibrational spectra of $Cs_2NaCe(NO_2)_6$ at 295 K. (T_h Sym: T_h symmetry irrep; Type: major contribution; sym str: symmetric stretch; antisym: antisymmetric; sciss: scissor; asym: asymmetric; s: strong; vw: very weak; vs: very strong; mw medium weak.

T_h Sym	Type	Energy (cm^{-1})	
		IR	Raman
A_g	N-O sym str		1334s
T_u	N-O antisym str	1329vw	
T_g	N-O str		1246vw
T_u	N-O str	1234vs	
A_g	NO_2 sciss		835vs
E_g	NO_2 sciss		831vs
T_u	NO_2 sciss	828ms	
T_g	NO_2 wag		294w
A_g	Ce-O sym str		236vw
T_g	NO_2 rock		198mw
E_g	Ce-NO_2 str		
T_g	NO_2 asym bend		148s
T_g	CeO_2 bend		127s

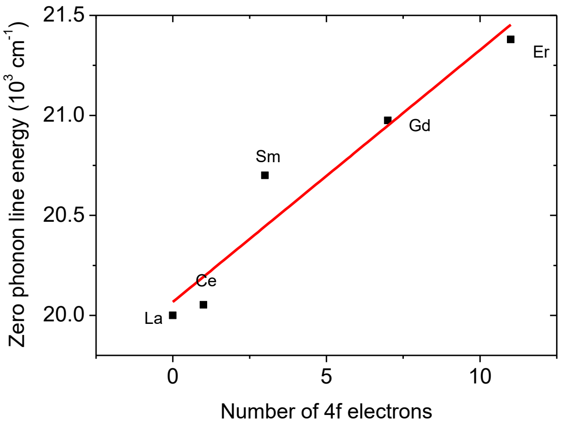

Figure. S6. Trend in NO_2^- zero phonon line energy for $Cs_2NaLn(NO_2)_6$ series. Data for La, Sm, Gd and Er measured from Fig. 2 (12 K absorption spectra) in ref. S1 and Ce this work. The fitting is a guide to the eye: $y = (20067 \pm 117) + (126 \pm 20)\,x$.

Table. S6. Ab initio crystal field parameters for Ln^{3+} in $Cs_2NaLn(NO_2)_6$ and $5d^1$ (1,2) crystal field splitting of Ce^{3+}.

Parameter	Value (cm^{-1})		
	Ce^{3+}	Pr^{3+}	Yb^{3+}
B_{40}	121	381	352
B_{60}	-223	-235	-112
B_{62}	932	1217	813
B_{40}(5d)	9715		
5d CF splitting	4625		

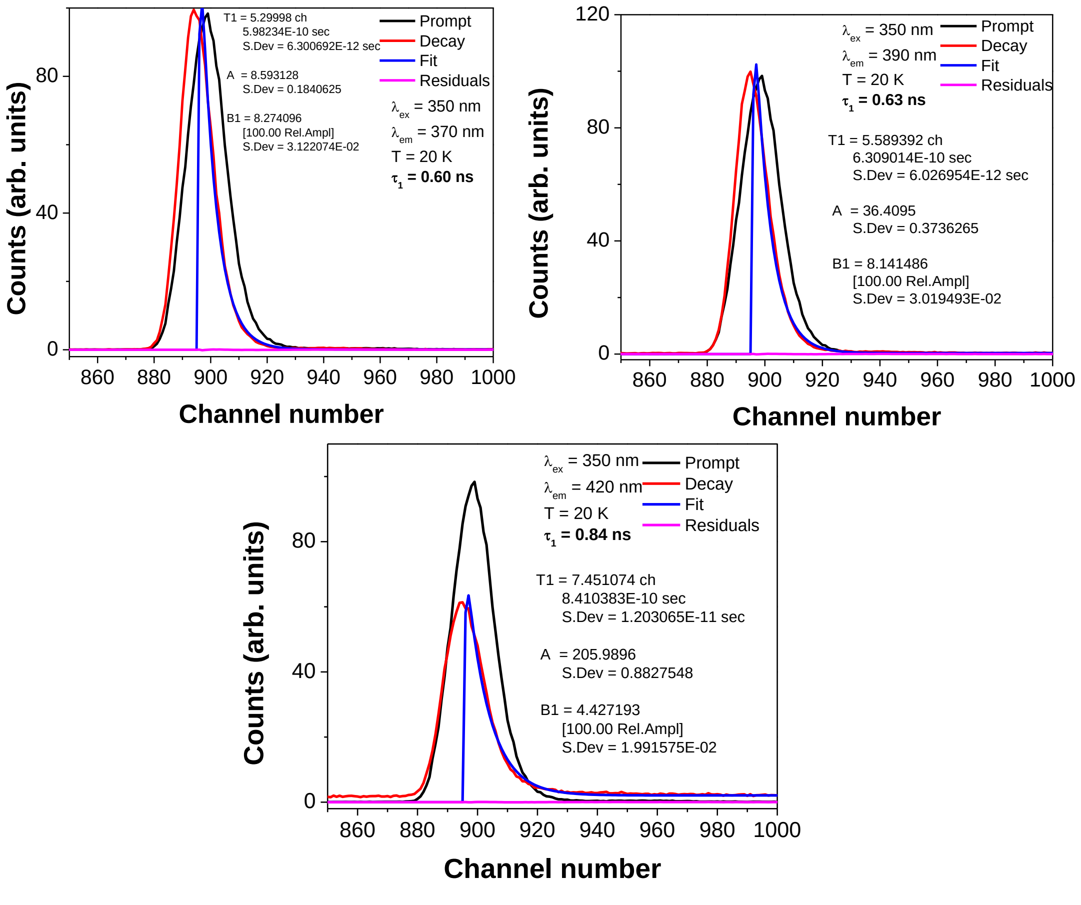

Figure. S7. Measurements at various wavelengths of $S_1 \rightarrow S_0$ singlet emission lifetime of $Cs_2NaLa(NO_2)_6$ at 20 K.

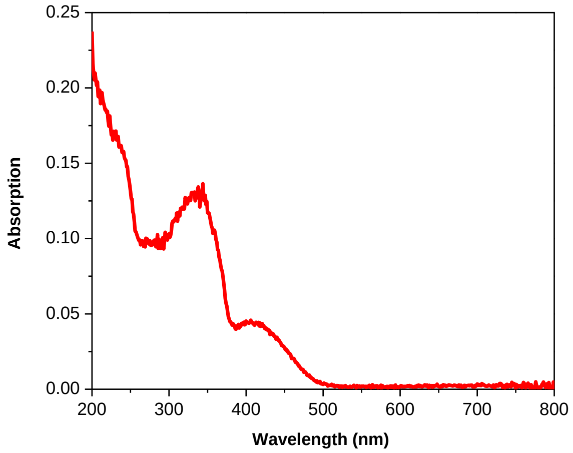

Figure. S8. Room temperature diffuse reflection spectrum of $Cs_2NaCe(NO_2)_6$.

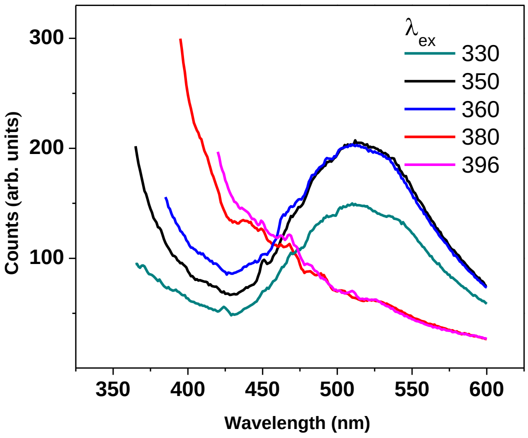

Figure. S9. 10 K emission spectra of $Cs_2NaCe(NO_2)_6$ using various excitation wavelengths.

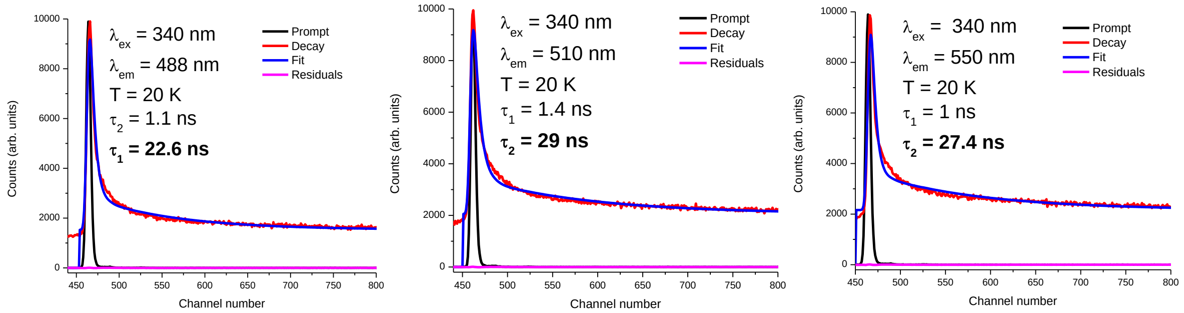

Figure. S10. Measured Ce^{3+} lifetime at 20 K for various emission wavelengths of $Cs_2NaCe(NO_2)_6$. The lifetime τ_1 refers to the excitation pulse.

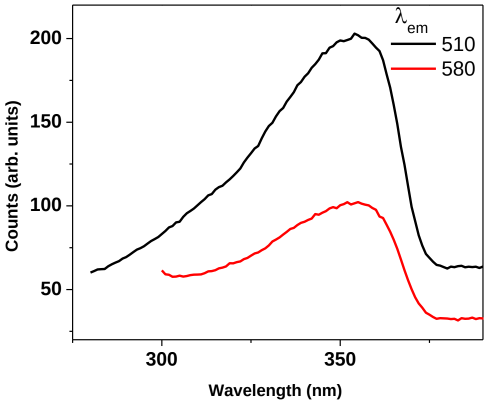

Figure. S11. 10 K excitation spectrum of $Cs_2NaCe(NO_2)_6$ monitoring two emission wavelengths.

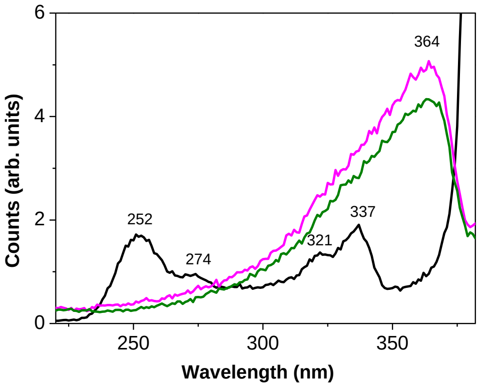

Figure. S12. 20 K excitation spectra of $Cs_2NaCe(NO_2)_6$ monitoring NO_2^- emission at 420 nm (black) and Ce^{3+} emission at 546 nm (pink) and 571 nm (green).

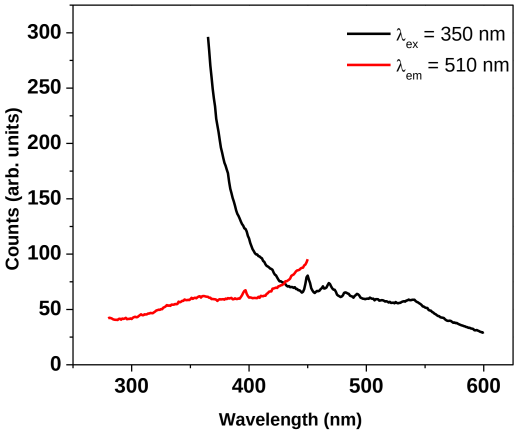

Figure. S13. 100 K excitation and emission spectra of $Cs_2NaCe(NO_2)_6$. The peaks are due to xenon lamp lines.

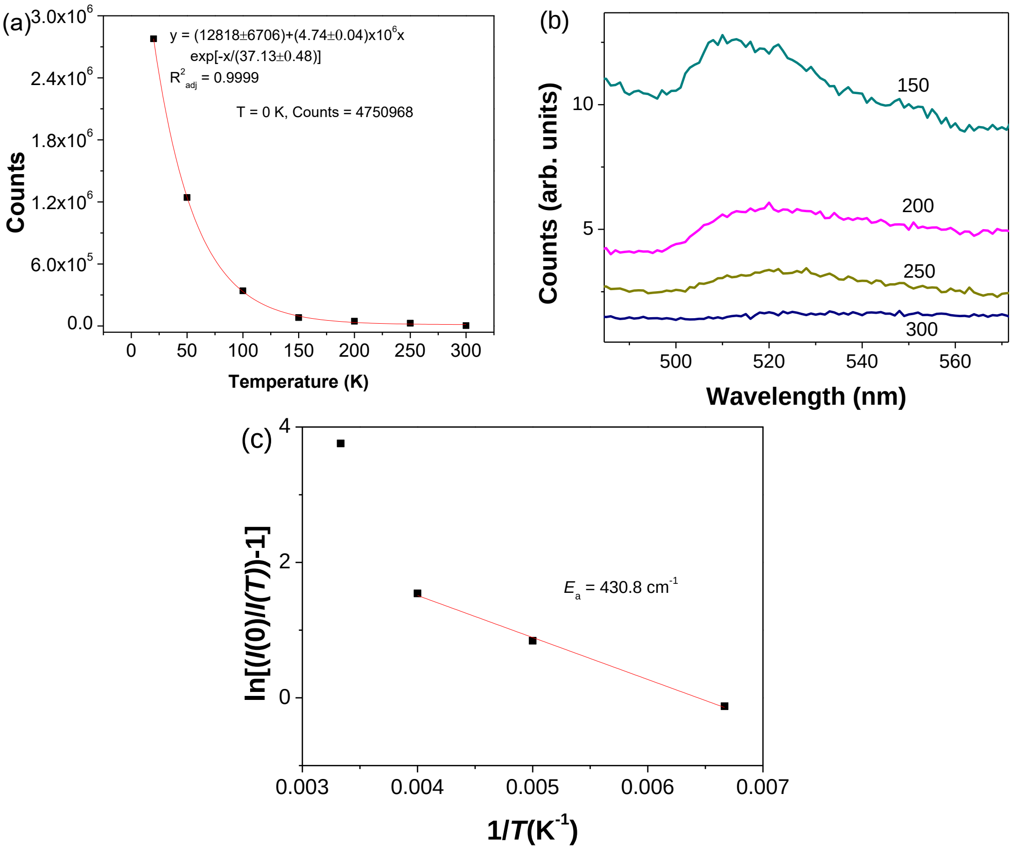

Figure. S14. (a) Integrated 5d – 4f emission spectra of Ce^{3+} in $Cs_2NaCe(NO_2)_6$ under 333 nm excitation at different temperatures; (b) Emission spectra between 490-560 nm for $Cs_2NaCe(NO_2)_6$ under 333 nm excitation from 150 K to 300 K; (c) Arrhenius plot for the temperature range from 150-250 K.

References

1 Kirschner, A. V. *et al*. Spectroscopy of hexanitritoelpasolite crystals: the effect of the rare-earth ion on the progressions in the nitrite vibration. *Spectrochim. Acta. A.* **54**, 2045-2049 (1998).

2 Roser, M. R. & Corruccini, L. R. Magnetic susceptibilities of rare-earth ions in an unusual tetrahedral site. *Phys. Rev. B.* **41**, 2359-2368 (1990).

3 Tanner, P. A., Li, W. Y. & Ning, L. X. Electronic spectra and crystal-field analysis of europium in hexanitritolanthanate systems. *Inorg. Chem.* **51**, 2997-3006 (2012).

4 Li, W. Y., Ning, L. X., Faucher, M. D. & Tanner, P. A. Experimental and theoretical studies of the vibrational and electronic spectra of a lanthanide ion at a site of T_h symmetry: Pr^{3+} in $Cs_2NaPr(NO_2)_6$. *Inorg. Chem.* **50**, 9004-9013 (2011).

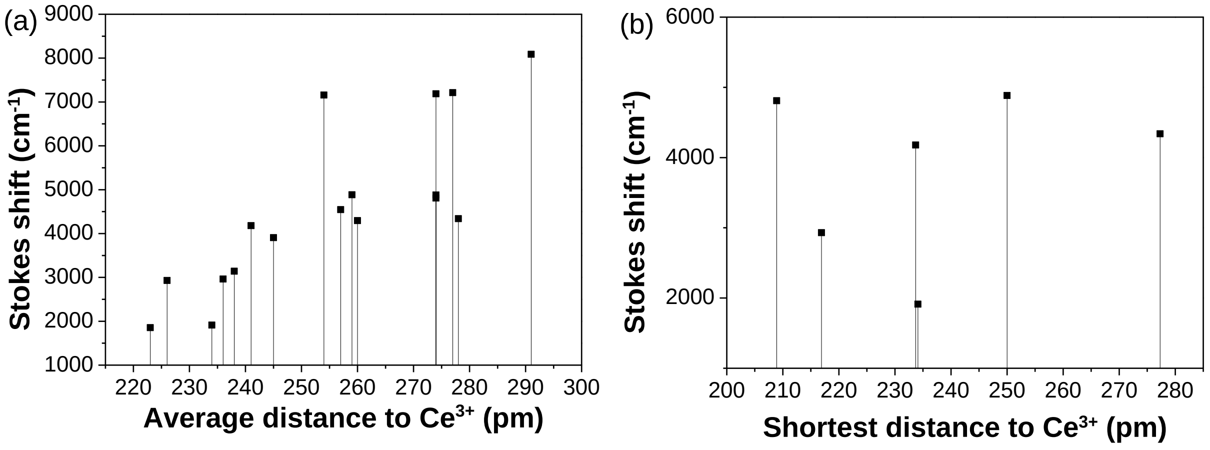

Figure. S15 Plot of Stokes shift for Ce-O systems against (a) average Ce-O distance and (b) shortest Ce-O distance from literature data, Table S7 and this work.

Table. S7. Stokes shifts and physical parameters of cerium-oxygen systems.

Compound	Coordination number	Polyhedron: Point symmetry	Ce-Ligand distance pm (shortest: average)	λexc (nm)	λem (nm)	Stokes shift (cm^{-1})	Ref.
$CaCO_3$	6	Octahedron:C_{3i} 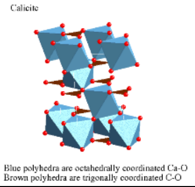	-:236	313	345	2963	1
$Lu_2Si_2O_7$	6	Octahedron: C_2	-:223	355	380	1853	2
$GdAl_3(BO_3)_4$	6	Trigonal:D_3 JCPDS No. 83-1907 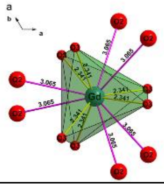	234.1:234.1	323	342	1913	3
$Na_3LuSi_3O_9$	6	Orthorhombic: $P2_12_12_1$(space group) 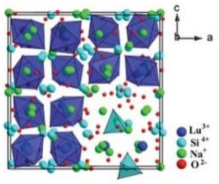 90% Ce^{3+} ions enter in the Lu1 and Lu4 sites (29% in Lu1 site and 61% in Lu4 site) and the remaining mostly embedded in Lu3 site	200.98:225.08 (Lu1 29%) 208.77:222.67 (Lu3 10%) 216.88:226.38 (Lu4 61%)	350	390	2930	4
$LiYSiO_4$	6	Octahedron ICSD 75538 Ce^{3+} substitutes Y^{3+} located at the center of a distorted octahedron		348	400	3736	5
$Ba_2Gd(BO_3)_2Cl$	7	Monoclinic: C_s JCPDS 79-0967 The Ln positions are 7 coordinated by O with site symmetry C_s. 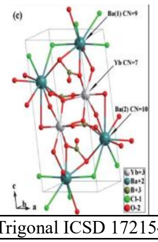		354	415	4153	6-7
$Ca_3Ln(AlO)_3(BO_3)_4$	7	Trigonal ICSD 172154	233.7:241.3	350	410	4181	8

		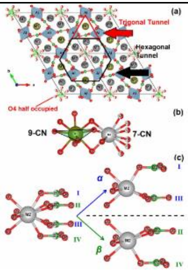 Ce^{3+} ions occupy the 7-fold coordinated M2 site					
$Y_3Al_5O_{12}$	8	Octahedral:D_2<br>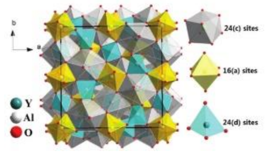	-:238	458	535	3142	9-11
$Ca_3Sc_2Si_3O_{12}$	8	Dodecahedra<br>ICDD 72-1969<br>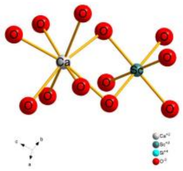<br>Ce^{3+} replaces the Ca^{2+} position	-:256.6	440	550	4545	12 13-15
$Ca_3Sc_2Si_3O_{12}$	8	Dodecahedral<br>ICDD 72-1969<br>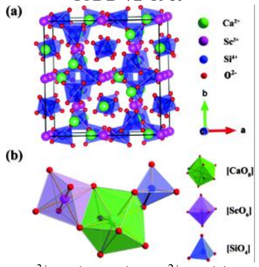 Ce^{3+} replaces the Ca^{2+} position	-:244.5 (x=1)<br>$Ca_{2.97-x}Y_xSc_{2-x}Mg_xSi_3O_{12}$:$Ce_{0.03}$	450	546	3907	14
$Ca_3Sc_2Si_3O_{12}$	8	Dodecahedral<br>ICDD 72-1969		447	510	2763	15
$Ca_3Hf_2SiAl_2O_{12}$	8	Cubic<br>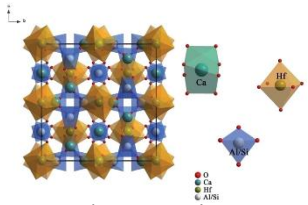 Ce^{3+} occupies Ca^{2+} site		400	457	3118	16
$NaBaPO_4$	8	Trigonal: $P3m1$<br>JCPDs 33-1210<br>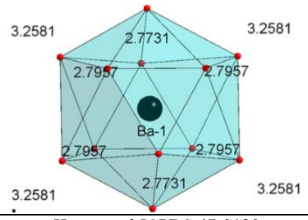	277.31：278	324	377	4339	17
Li_2SrSiO_4	8	Hexagonal JCPDS 47-0120<br>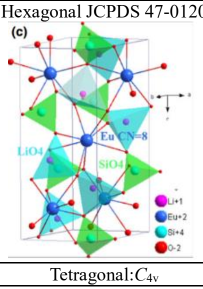		360	428	4413	18
$CaYAlO_4$	9	Tetragonal:C_{4v}	-:254	360	485	7159	19

		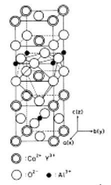<br>The Ca^{2+}/Y^{3+} ions are surrounded by nine nearest-neighbour oxygen ligands. Ce^{3+} ions occupy Ca^{2+}/Y^{3+} sites					
$LaBO_3$	9	Orthorhombic: C_s<br>JCPDS 12-0762	-:260	329	383	4295	20-21
$LiBaPO_4$	9	Hexagonal JCPDS 14-0270<br>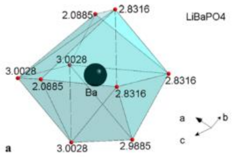	208.85:274.1	380	468	4810	17
$LaPO_4$	9	Monoclinic: $P2_1/n$<br>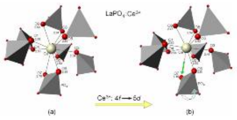	250：259	273	315	4884	22
$LaPO_4$	9	Monoclinic		254	320	8120	23
$NaSrBO_3$	9	Monoclinic<br>ICSD-172420<br>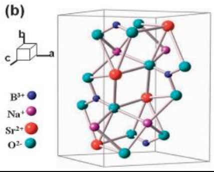		345	424	5401	24
$SrSO_4$	10	Orthorhombic: *P*nma<br>JCPDS 80-0523	-：274	267	307	4880	25-26
$BaSO_4$	10	Orthorhombic: *P*nma	-：288				26
$La_2Be_2O_5$	10	Irregular: C_1	-:291	354	496	8088	27-28
$LaScO_3$	12	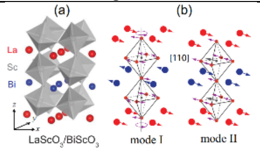		323	429	7700	29-30
$LaMgAl_{11}O_{19}$	12	Hexagonal: D_{3h}<br>JCPDS 26-0873<br>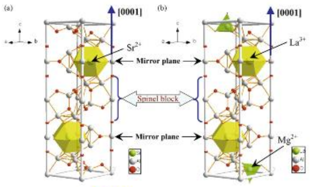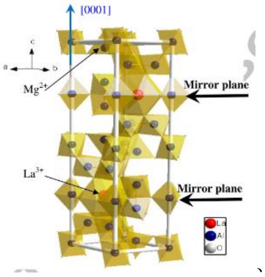<br>La^{3+} ions are located in the intermediate mirror plane having 12 coordination	-:274	270	335	7186	31-34
$CaHfO_3$	12			334	431	6700	29
$LaScO_3$	12			323	429	7700	29
$GdScO_3$	12			349	429	5350	29
$SrA1_{12}O_{19}$	12	Hexagonal	-:277	260	320	7212	35

		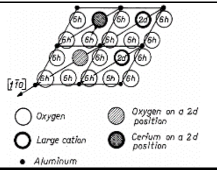					

1. Blasse, G.; Aguilar, M., Luminescence of natural calcite ($CaCO_3$). *Journal of Luminescence* **1984,** *29* (5), 239-241.
2. Pauwels, D.; Masson, N. L.; Viana, B.; Kahn-Harari, A.; Loef, E. V. D. v.; Dorenbos, P.; Eijk, C. W. E. v., A novel inorganic scintillator: $Lu_2Si_2O_7$:Ce^{3+} (LPS). *IEEE Transactions on Nuclear Science* **2000,** *47* (6), 1787-1790.
3. He, J.; Shi, R.; Brik, M. G.; Dorenbos, P.; Huang, Y.; Tao, Y.; Liang, H., Luminescence and multi-step energy transfer in $GdAl_3(BO_3)_4$ doped with Ce^{3+}/Tb^{3+}. *Journal of Luminescence* **2015,** *161*, 257-263.
4. Zhou, J.; Pan, F.; Zhong, J.; Liang, H.; Su, Q.; Moretti, F.; Lebbou, K.; Dujardin, C., Luminescence properties of $Na_3LuSi_3O_9$:Ce^{3+} as a potential scintillator material. *RSC Advances* **2015,** *5* (124), 102477-102480.
5. Shi, R.; Xu, J.; Liu, G.; Zhang, X.; Zhou, W.; Pan, F.; Huang, Y.; Tao, Y.; Liang, H., Spectroscopy and Luminescence Dynamics of Ce^{3+} and Sm^{3+} in $LiYSiO_4$. *The Journal of Physical Chemistry C* **2016,** *120* (8), 4529-4537.
6. Schipper, W. J.; Blasse, G., Luminescence in the chloroborates $Ba_2Ln(BO_3)_2Cl$. *Journal of Alloys and Compounds* **1994,** *203*, 267-269.
7. Jing, H.; Guo, C.; Zhang, G.; Su, X.; Yang, Z.; Jeong, J. H., Photoluminescent properties of Ce^{3+} in compounds $Ba_2Ln(BO_3)_2Cl$ (Ln = Gd and Y). *Journal of Materials Chemistry* **2012,** *22* (27), 13612-13618.
8. Wen, D.; Kato, H.; Kobayashi, M.; Yamamoto, S.; Mitsuishi, M.; Kakihana, M., Site occupancy and luminescence properties of $Ca_3Ln(AlO)_3(BO_3)_4$:Ce^{3+},Tb^{3+},Mn^{2+} (Ln = Y, Gd). *Journal of Materials Chemistry C* **2017,** *5* (18), 4578-4583.
9. Robbins, D. J., The Effects of Crystal Field and Temperature on the Photoluminescence Excitation Efficiency of Ce^{3+} in YAG. *Journal of The Electrochemical Society* **1979,** *126* (9), 1550-1555.
10. Holloway, W. W.; Kestigian, M., On the fluorescence of cerium-activated garnet crystals. *Physics Letters A* **1967,** *25* (8), 614-615.
11. Xia, Z.; Meijerink, A., Ce^{3+}-Doped garnet phosphors: composition modification, luminescence properties and applications. *Chem Soc Rev* **2017,** *46* (1), 275-299.
12. Zhou, L.; Zhou, W.; Pan, F.; Shi, R.; Huang, L.; Liang, H.; Tanner, P. A.; Du, X.; Huang, Y.; Tao, Y.; Zheng, L., Spectral Properties and Energy Transfer of a Potential Solar Energy Converter. *Chem Mater* **2016,** *28* (8), 2834-2843.
13. Liu, Y.; Zhuang, W.; Hu, Y.; Gao, W.; Hao, J., Synthesis and luminescence of sub-micron sized $Ca_3Sc_2Si_3O_{12}$:Ce green phosphors for white light-emitting diode and field-emission display applications. *Journal of Alloys and Compounds* **2010,** *504* (2), 488-492.
14. Pan, F.; Zhou, M.; Zhang, J.; Zhang, X.; Wang, J.; Huang, L.; Kuang, X.; Wu, M., Double substitution induced tunable luminescent properties of $Ca_{3-x}Y_xSc_{2-x}Mg_xSi_3O_{12}$:$Ce^{3+}$ phosphors for white LEDs. *Journal of Materials Chemistry C* **2016,** *4* (24), 5671-5678.
15. Suzuki, Y.; Kakihana, M.; Shimomura, Y.; Kijima, N., Synthesis of $Ca_3Sc_2Si_3O_{12}$:Ce^{3+} phosphor by hydrothermal Si alkoxide gelation. *Journal of Materials Science* **2008,** *43* (7), 2213-2216.
16. Ding, X.; Geng, W.; Wang, Q.; Wang, Y., Structure, luminescence property and abnormal energy transfer behavior of color-adjustable $Ca_3Hf_2SiAl_2O_{12}$:Ce^{3+},Mn^{2+} phosphors. *RSC Advances* **2015,** *5* (119), 98709-98716.
17. Wei, D.; Huang, Y.; Zhang, S.; Yu, Y. M.; Seo, H. J., Luminescence spectroscopy of Ce_{3+}-doped $ABaPO_4$ (A=Li, Na, K) phosphors. *Applied Physics B* **2012,** *108* (2), 447-453.
18. Chen, J.; Guo, C.; Yang, Z.; Li, T.; Zhao, J., Li_2SrSiO_4:Ce^{3+}, Pr^{3+} Phosphor with Blue, Red, and Near-Infrared Emissions Used for Plant Growth LED. *Journal of the American Ceramic Society* **2016,** *99* (1), 218-225.
19. Kodama, N.; Yamaga, M.; Henderson, B., Inhomogeneous broadening of the Ce^{3+} luminescence in $CaYAlO_4$. *Journal of Physics: Condensed Matter* **1996,** *8* (19), 3505.
20. Blasse, G.; van Vliet, J. P. M.; Verwey, J. W. M.; Hoogendam, R.; Wiegel, M., Luminescence of Pr^{3+} in scandium borate ($ScBo_3$) and the host lattice dependence of the stokes shift. *Journal of Physics and Chemistry of Solids* **1989,** *50* (6), 583-585.
21. Guerbous, L.; Seraiche, M.; Krachni, O., Photoluminescence and electron-vibrational interaction in $4f^n$–5d states of Ce^{3+} or Pr^{3+} ions doped $LnBO_3$ (Ln=Lu, Y, La) orthoborates materials. *Journal of Luminescence* **2013,** *134*, 165-173.
22. Bagatur'yants, A. A.; Iskandarova, I. M.; Knizhnik, A. A.; Mironov, V. S.; Potapkin, B. V.; Srivastava, A. M.; Sommerer, T. J., Energy level structure of 4f5d states and the Stokes shift in $LaPO_4$:Pr^{3+}: A theoretical study. *Physical Review B* **2008,** *78* (16), 165125.
23. Hoffman, M. V., Effect of Thorium on Ce^{3+} Phosphors. *Journal of The Electrochemical Society* **1971,** *118* (9), 1508-1510.
24. Xin, M.; Tu, D.; Zhu, H.; Luo, W.; Liu, Z.; Huang, P.; Li, R.; Cao, Y.; Chen, X., Single-composition white-emitting $NaSrBO_3$:Ce^{3+},Sm^{3+},Tb^{3+} phosphors for NUV light-emitting diodes. *Journal of Materials Chemistry C* **2015,** *3* (28), 7286-7293.
25. Sun, J.; Sun, G.; Du, H., Facile room temperature synthesis of $SrSO_4$:RE (RE=Sm^{3+}, Tb^{3+}, Ce^{3+}) microrods via a precipitation method and its luminescence properties. *Mater Res Bull* **2013,** *48* (10), 3939-3942.
26. van der Kolk, E.; Dorenbos, P.; Vink, A. P.; Perego, R. C.; van Eijk, C. W. E.; Lakshmanan, A. R., Vacuum ultraviolet excitation and emission properties of Pr^{3+} and Ce^{3+} in MSO_4 (M=Ba,Sr, and Ca) and predicting quantum splitting by Pr^{3+} in oxides and fluorides. *Physical Review B* **2001,** *64* (19), 195129.
27. Ogorodnikov, I. N.; Pustovarov, V. A., Linear optical, luminescence and electronic properties of the $La_2Be_2O_5$ laser crystals doped with Ce^{3+} or Eu^{3+}. *Journal of Luminescence* **2015,** *162*, 50-57.
28. Dorenbos, P., 5d-level energies of Ce^{3+} and the crystalline environment. IV. Aluminates and "simple" oxides. *Journal of Luminescence* **2002,** *99* (3), 283-299.
29. Setlur, A.; Happek, U., Luminescence of Ce^{3+} in the Scandate Perovskites. *ECS Transactions* **2009,** *16* (31), 51-56.
30. Gou, G.; Rondinelli, J. M., Ferroelectrics: Piezoelectricity Across a Strain-Induced Isosymmetric Ferri-to-Ferroelectric Transition. *Advanced Materials Interfaces* **2014,** *1* (5).
31. Viana, B.; Aka, G.; Vivien, D.; Lejus, A. M.; Théry, J.; Derory, A.; Bernier, J. C.; Garapon, C.; Boulon, G., Absorption, fluorescence, and electron spin resonance investigation of tervalent cerium activated $LaMgAl_{11}O_{19}$. *Journal of Applied Physics* **1988,** *64* (3), 1398-1404.
32. Min, X.; Fang, M.; Huang, Z.; Liu, Y. g.; Tang, C.; Qian, T.; Wu, X., Synthesis and luminescence properties of nitrided lanthanum magnesium hexaluminate $LaMgAl_{11}O_{19}$ phosphors. *Ceramics International* **2014,** *40* (3), 4535-4539.
33. Chen, X.; Zhang, Y.; Zhong, X.; Xu, Z.; Zhang, J.; Cheng, Y.; Zhao, Y.; Liu, Y.; Fan, X.; Wang, Y.; Ma, H.; Cao, X., Thermal cycling behaviors of the plasma sprayed thermal barrier coatings of hexaluminates with magnetoplumbite structure. *Journal of the European Ceramic Society* **2010,** *30* (7), 1649-1657.
34. Chen, X.; Cao, X.; Zou, B.; Gong, J.; Sun, C., Corrosion of lanthanum magnesium hexaaluminate as plasma-sprayed coating and as bulk material when exposed to molten V_2O_5-containing salt. *Corrosion Science* **2015,** *91*, 185-194.

35. Stevels, A. L. N., Ce^{3+} Luminescence in Hexagonal Aluminates Containing Large Divalent or Trivalent Cations. *Journal of The Electrochemical Society* **1978,** *125* (4), 588-594.
